# Transient Elastography Increases Readiness for Change in Inpatients With Alcohol Use Disorder: The ELISA Pilot Study

**DOI:** 10.1111/adb.70043

**Published:** 2025-06-23

**Authors:** Stephanie M. Rutledge, Rohit R. Nathani, Patricia Miguez Arosemena, Daniel Suter, David Lehman, Timothy Brennan, Gene Y. Im

**Affiliations:** ^1^ Division of Liver Diseases Icahn School of Medicine at Mount Sinai New York New York USA; ^2^ Division of Gastroenterology and Hepatology Weill Cornell Medical College New York New York USA; ^3^ Department of Medicine Mount Sinai Morningside and West New York New York USA; ^4^ Department of Psychiatry, Addiction Institute of New York Icahn School of Medicine at Mount Sinai New York New York USA; ^5^ Center for Liver Disease and Transplantation Columbia University Vagelos College of Physicians and Surgeons New York New York USA

**Keywords:** alcohol‐associated liver disease, fibrosis, motivation, opportunistic intervention, opportunistic screening

## Abstract

Opportunistic interventions (OIs) are health events facilitated by healthcare providers through education that can motivate individuals to adopt risk‐reducing behaviours. Our aim was to evaluate transient elastography (TE) as an OI in patients with AUD by assessing changes in validated psychometric scores (PS) of alcohol insight and readiness for change. In this prospective, proof‐of‐concept pilot study, patients with AUD without TE in the past year were enrolled from an inpatient addiction unit. At baseline, three PS assessing insight and readiness to change were administered: Hanil Alcohol Insight Scale (HAIS), revised Readiness Ruler (RR) and Stages of Change Readiness and Treatment Eagerness Scale (SOCRATES‐8A). TE was performed, interpreted, and followed by repeat PS testing. The primary outcome was change in PS. Secondary outcomes were prevalence of fibrosis and steatosis on TE, alcohol use and linkage to hepatology care. From 4 January 2022 to 4 January 2023, 23 patients were enrolled: mean age: 51 years (SD ± 12), 74% male, 61% White and all with severe AUD, and mean of 20 (± 9) daily drinks, 286 g (± 127 g) or 35.7 (± 15.9) units of alcohol, for a median of 14 years (IQR 10–21.5). After TE, there were significant increases in revised RR and SOCRATES‐8A from 5 to 8.6 (± 2.1, *p* < 0.01) and 81.5 to 85.0 (± 8.0, *p* = 0.04), respectively, indicating improved motivation and readiness for change. HAIS did not change: 11.1–11.0 (± 3, *p* = 0.36). Cirrhosis and steatosis grade ≥ 2 were detected in 4/23 (17%) and 8/23 (35%), respectively. In this pilot study, performing and interpreting results of TE to inpatients with AUD increase readiness for change and efficiently detects advanced fibrosis.

## Graphical abstract

In this prospective, proof‐of‐concept pilot study of transient elastography as an opportunistic intervention in patients admitted to an addiction unit by assessing changes in validated psychometric scores of alcohol insight and readiness for change. Opportunistic interventions are health events facilitated by healthcare providers that can motivate individuals to adopt risk‐reducing behaviours. After transient elastography, there were significant increases in readiness for change scores, but no change in insight.

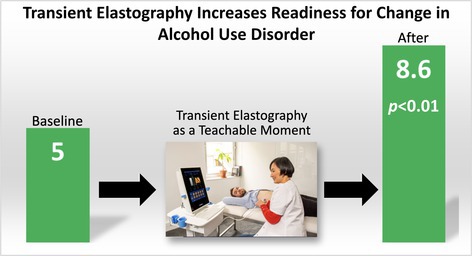

## 1 Introduction

Alcohol‐associated liver disease (ALD) is the leading cause of cirrhosis‐related mortality in the United States (US) and in many parts of the world [1, 2, 3]. Alcohol use has increased over the past two decades in the US and was exacerbated by the COVID‐19 pandemic [4, 5]. A subsequent rise in ALD and ALD‐related mortality was seen, especially in young adults [6]. These epidemiologic trends highlight the lack of advancements in early detection and effective therapies for alcohol use disorder (AUD) and ALD.

Although psychosocial interventions such as community alcohol rehabilitation programmes and psychotherapy are the cornerstones of AUD treatment, relapse is common [7]. A systematic review of psychosocial interventions for AUD in patients with chronic liver disease demonstrated that only an integrated approach combining psychotherapy with comprehensive medical care increased alcohol abstinence [8]. This combined intervention may have leveraged the integrated patient experience as a series of opportunistic interventions (OIs), sometimes called teachable moments [9]. OIs are health events facilitated by healthcare providers through ‐ timely education that can motivate individuals to adopt risk‐reducing behaviours [10].

Motivation is affected by external and internal factors, including perceived benefits of abstinence and perceived severity of harm [11]. Motivation predicts engagement with addiction treatment [12, 13, 14]. Additionally, life stressors and experiencing medical illness can be motivators for change [15]. New awareness of liver disease gained through an OI may increase motivation via ‘self‐concept’ (an individual's perception of their social role and responsibilities and acceptability of their behaviour to society) [10]. Experiencing physical symptoms of liver disease may transform an abstract health risk into a tangible reality and can serve as a powerful catalyst for behavioural change. However, not all patients stop drinking after a diagnosis of ALD. There may be perceived futility of abstinence, and the subset of patients who develop ALD typically has a long history of severe AUD [7]. Additionally, patients with AUD and ALD require interdisciplinary management with close communication and collaboration between specialists. Adequately comanaging both conditions can lead to successful outcomes in this medically and psychiatrically complex population. Ideally, patients with AUD or alcohol misuse would be diagnosed and treated at an early stage of liver disease.

In patients with AUD at risk of liver disease, transient elastography (TE), a noninvasive, medical imaging test commonly used in clinical practice to risk stratify liver disease, has potential as an OI. TE is inexpensive and simple to use and takes less than 5 min to perform, and the machine is often colocated in outpatient gastroenterology/hepatology clinics. It utilizes shear wave ultrasonography that provides a visual and numerical result of liver stiffness (fibrosis) and fat content (steatosis) that correlates well with clinical outcomes. TE is a well‐validated method to determine the presence of significant liver fibrosis noninvasively (instead of liver biopsy) per practice guidelines [16]. We hypothesize that TE, when performed and results interpreted in real‐time, is an OI that can increase patient motivation towards abstinence in combination with AUD treatment. Our aim was to evaluate TE as a potential OI in patients with AUD admitted to an addiction unit by assessing changes in psychometric scores (PSs) of alcohol insight and readiness for change.

## 2 Methods

### 2.1 Study Design

This was a prospective, investigator‐initiated, proof‐of‐concept pilot study to determine whether TE performance and interpretation improves alcohol insight and readiness for change in patients with AUD at risk of ALD: the Evaluating LIver Stiffness and steatosis as an OI in patients with AUD (ELISA) study. Within 7 days of admission, subjects were administered a questionnaire, followed by three psychometric scores (PS): the Hanil Alcohol Insight Scale (HAIS) [17], the revised Readiness Ruler tool (RR) [18] and the Stages of Change Readiness and Treatment Eagerness Scale (SOCRATES‐8A) (Figures S1–S4) [19]. TE was then performed (FibroScan, device support provided by Echosens, Paris, France) and results given to the subject in real‐time via prescripted verbal interpretations by a physician with specific interest and training in both AUD and ALD (Table S1). Because even nonsevere alcohol‐associated hepatitis (AH) can increase liver stiffness when measured by TE, TE interpretations were adjusted based on bloodwork (AST and bilirubin) when applicable (Table S2) [20]. Together, these activities constituted TE as an OI. HAIS, revised RR and SOCRATES‐8A were then immediately readministered to the subject to determine score changes post‐TE. All patients admitted to the addiction unit received outpatient AUD treatment referrals upon discharge, which is standard of care for the unit. Subjects with liver stiffness measurements (LSM) ≥ 8.8 kPa (F3 or higher) were provided referrals to hepatology care per practice guidelines [16]. Our team facilitated a new patient visit with the study PI (hepatologist). Within 1 month of the TE results, administrative staff called the patient to offer an appointment. We measured rates of successful scheduling and completion of this visit.

### 2.2 Study Population

We included adults (≥ 18 years old) admitted to an inpatient addiction unit in New York City with a diagnosis of AUD (Table S3). We excluded patients with TE performed in the past 1 year; non‐English speakers; pregnant patients; uncontrolled hepatic encephalopathy; uncontrolled concomitant psychiatric disorder, CIWA‐AR score > 20 (severe); and with inability to perform TE due to body habitus or technical issues. All patients provided informed consent. Research was approved by the Mount Sinai institutional review board.

### 2.3 Baseline Assessments

All patients completed a baseline assessment within 7 days of admission including sociodemographic, clinical and laboratory information. The Diagnostic and Statistical Manual of Mental Disorders (DSM‐V) criteria was used for the diagnosis of AUD, and the Alcohol Use Disorders Identification Test‐C (AUDIT‐C) was used to characterize alcohol use (Figure S5). Three validated psychometric instruments were used to assess changes in alcohol insight and readiness for change. HAIS is a 20‐item questionnaire that examines subjects' acceptance or denial of their AUD diagnosis with scores ranging from 20 to −20. Higher scores indicate higher level of insight: −20 to +3 (poor insight), 4–15 (fair insight) and 16–20 (good insight). The RR is a single question instrument that assesses a person's willingness or readiness to change by determining where they are on the continuum between ‘not prepared to change’ and ‘already changing’ (continuous scale from 0 to 10) [18]. Because individuals admitted to an addiction unit may already have high baseline RR scores, the RR was revised to have a preset score of 5 for motivation to stop drinking pre‐TE, with subjects asked to choose a score from 0 to 10 for readiness to change drinking post‐TE. SOCRATES‐8A is a 19‐item questionnaire that assesses motivation for change in patients with alcohol misuse [19]. It provides quantitative measures of three domains (recognition, ambivalence and taking steps) and a composite motivation score ranked as low, medium or high relative to people already presenting for alcohol treatment.

### 2.4 Outcomes

The primary outcome was change from baseline HAIS, revised RR and SOCRATES‐8A scores assessed after TE performance and interpretation. Secondary outcomes were prevalence of fibrosis and steatosis on TE, change in alcohol use and linkage to hepatology care rates in subjects with F3 fibrosis or higher. Follow‐up phone calls were made at 1, 3, 6 and 12 months with alcohol use determined by self‐report. Comparisons of change in PS were made using two‐tailed, paired *T* tests with each patient serving as their own control. Analyses were performed using Stata 17 (College Station, TX).

## 3 Results

### 3.1 Subject Screening and Characteristics

From 4 January 2022 to 5 January 2023, 24 patients were screened; all were eligible and provided consent and were enrolled in the pilot study (Figure S6). One subject was subsequently excluded due to body habitus (high BMI) that precluded accurate TE measurement. Subject characteristics (*n* = 23) are outlined in Table 1, with mean age 51 years (standard deviation [SD] ± 12.1), 74% male and 61% White. All subjects were diagnosed with severe AUD, with a mean of 20.4 (± 9.1) daily drinks, 286 g (± 127 g) or 35.7 (± 15.9) units of alcohol, for a median of 14.0 (10–21.5) years and 18/23 (78%) had at least one prior failed alcohol rehabilitation attempt. The mean AUDIT‐C score was 11.6 (± 1.1, score range 0–12), consistent with hazardous drinking. Median AST and total bilirubin at admission were 35 U/L (IQR 20–70) and 0.5 mg/dL (IQR 0.35–0.65), respectively. Psychiatric comorbidities were present in 65%, most commonly depression (11/23, 48%), followed by anxiety (10/23, 43%) and posttraumatic stress disorder (5/23, 22%). Concurrent substance use was seen in 12/23 (52%) subjects: 6 cocaine and 6 marijuana.

**TABLE 1:** Patient characteristics.

Characteristic	Value
Age (years)—mean/SD	51 (12.1)
Sex	
Male	17 (74%)
Female	6 (26%)
Race/ethnicity	
White	14 (61%)
Black	4 (17%)
Hispanic	2 (9%)
Other	3 (13%)
BMI (kg/m^2^)—mean/SD	26.1 (3.9)
Currently employed	8 (35%)
Insurance status	
Private	15 (65%)
Medicare	4 (17%)
Medicaid	4 (17%)
Married or partner	3 (13%)
Days since last drink—median/IQR	6 (3–10)
Drinks/day prior to admission—mean/SD	20.4 (9.1)
Years of heavy drinking—median/IQR	14 (10–21.5)
Co‐morbid psychiatric diagnosis^a^	15 (65%)
Depression	11 (48%)
Anxiety	10 (43%)
PTSD	5 (22%)
Panic disorder	2 (9%)
Bipolar disorder	1 (4%)
Psychiatric medication use at admission	10 (43%)
Other substance use	12 (52%)
Current cigarette smoking	12 (52%)
Prior alcohol rehabilitation	
None	4 (17%)
1	4 (17%)
> 1	14 (61%)
Family history of alcohol use disorder	
None	6 (26%)
Immediate family member	13 (57%)
Extended family member	2 (9%)
Unknown	2 (9%)
Prior legal episodes from alcohol	
None	18 (78%)
1	2 (9%)
> 1	3 (13%)
Sodium (mEq/L)—mean/SD	140 (3)
Creatinine (mg/dL)—mean/SD	0.9 (2.1)
WBC (× 10^9^/L)—mean/SD	6.5 (1.7)
Platelets (× 10^9^/L)—mean/SD	224 (51)
Total bilirubin (mg/dL)—mean/IQR	0.5 (0.35–0.65)
AST (U/L)—median/IQR	35 (20–70)
ALT (U/L)—median/IQR	43 (14–71)
Albumin (mg/dL)—mean/SD	3.8 (0.3)
HCV antibody positive	1 (4%)
HBsAg positive	0
CAP (dB/m)—median/IQR	246 (221–296)
Steatosis grade on transient elastography	
0	3 (13%)
1	12 (52%)
2	2 (9%)
3	6 (26%)
LSM (kPA)—median/IQR	5.1 (3.8–9.4)
Fibrosis stage on transient elastography	
F0–F1	16 (70%)
F2	3 (13%)
F3	0
F4	4 (17%)

Abbreviations: ALT, alanine aminotransferase; AST, aspartate aminotransferase; CAP, controlled attenuation parameter; HBsAg, hepatitis B surface antigen; HCV, hepatitis C virus; IQR, interquartile range; LSM, liver stiffness measurement; PTSD, posttraumatic stress disorder; SD, standard deviation; WBC, white blood cell count.

^a^ Some subjects had more than one psychiatric diagnosis.

### 3.2 Psychometric Score Outcomes

The preintervention and postintervention PS are outlined in Table 2. Overall, the mean baseline HAIS score was 11.1 (± 3.4; range 9–19) and interpreted as ‘fair insight’, and as previously mentioned, the baseline revised RR score was preset at 5 (range 0–10). In contrast, the mean baseline SOCRATES‐8A score was 81.5 (± 8.4) and interpreted as ‘high’ readiness for change (> 80th centile compared to relative to people already presenting for alcohol treatment). Individual SOCRATES‐8A domain scores were ‘medium’ for ‘recognition’ and ‘ambivalence’ and ‘medium‐high’ for ‘taking steps’.

**TABLE 2:** Psychometric scores before and after transient elastography as an opportunistic intervention.

Psychometric score	Before TE Mean (SD)	Post‐TE as an OI Mean (SD)	Overall score change	*p* value
AUDIT‐C (Range: 0–12)	11.6 (1.1)	—	—	—
Hanil Alcohol Insight Scale (Range: −20 to 20)	11.1 (3.4)	11.0 (3.0)	−0.1	0.36
Revised Readiness Ruler (Range: 0–10)	5 (preset)	8.6 (2.1)	+3.6	**< 0.01**
SOCRATES‐8A (Range: 0–95)	81.5 (8.4)	85 (8.0)	+3.5	**0.04**
• Recognition domain (Range: 0–35)	32 (3.2)	33 (2.7)	+1	**0.02**
• Ambivalence domain (Range: 0–20)	15 (4.6)	15 (4.9)	0	0.17
• Taking Steps domain (Range: 0–40)	35 (3.5)	37 (2.8)	+2	**0.02**

*Note:* Bolded *p* values designate statistical significance (< 0.05).

Abbreviations: AUDIT‐C, Alcohol Use Disorder Identification Test‐Consumption; SD, standard deviation; SOCRATES‐8A, Stages of Change Readiness and Treatment Eagerness Scale; TE, transient elastography; TM, teachable moment.

**FIGURE 1:**
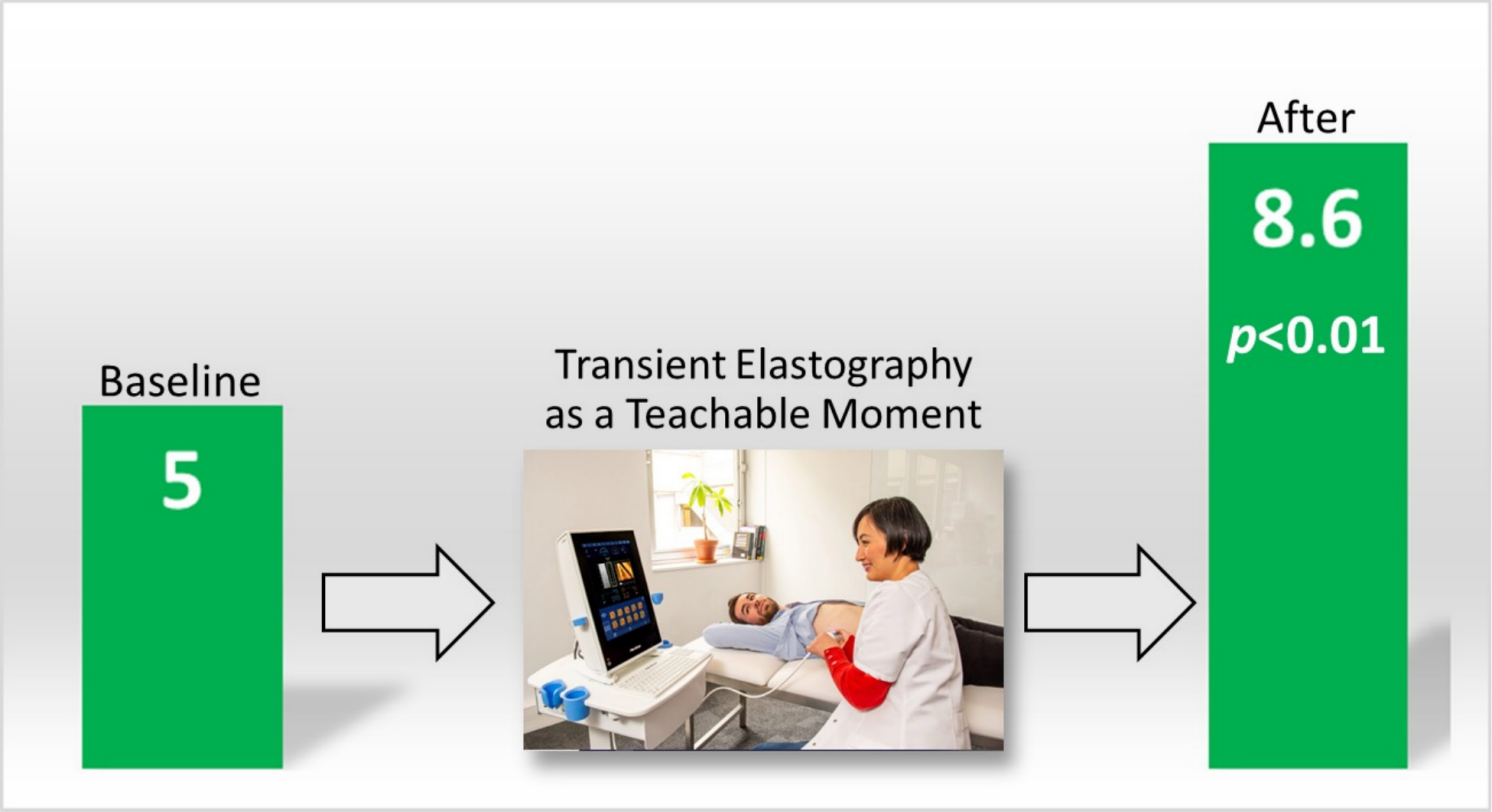
Change in Revised Readiness Ruler scores.

**FIGURE 2:**
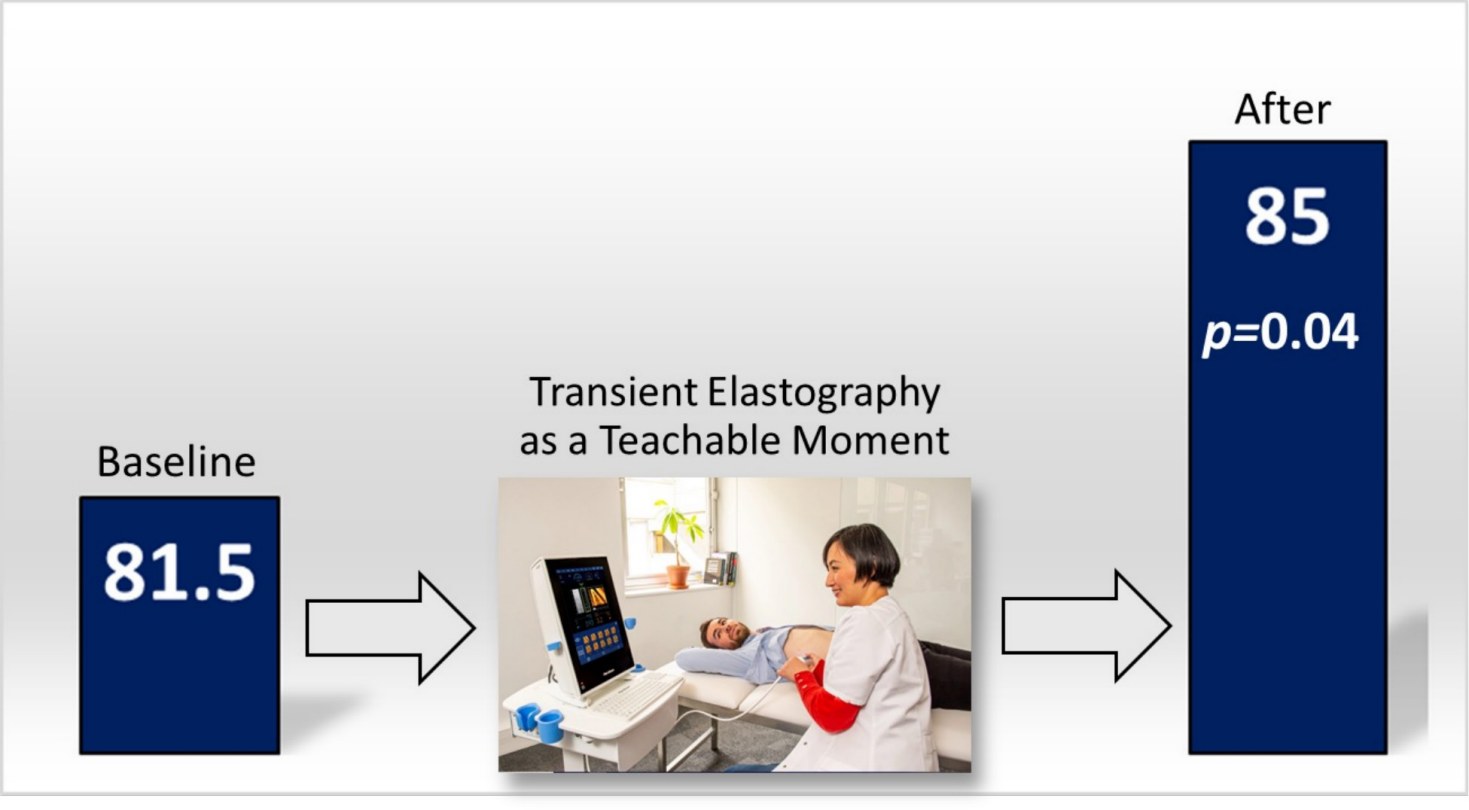
Change in SOCRATES‐8A scores.

After the TE as an OI, no change from baseline was noted for HAIS: 11.1–11.0 (± 3.0, *p* = 0.36). There were significant increases from mean baseline scores for revised RR and SOCRATES‐8A from 5 to 8.6 (± 2.1, *p* < 0.01) and 81.5 to 85.0 (± 8.0, *p* = 0.04), respectively (Figures 1 and 2). Further analysis of the SOCRATES‐8A domains demonstrated significant increases from mean baseline scores for two out of three domains: ‘recognition’ (32 ± 3.2 to 33 ± 2.7, *p* = 0.02) and ‘taking steps’ (35 ± 3.5 to 37 ± 2.8, *p* = 0.02) but not ‘ambivalence’ (15 ± 4.6 to 15 ± 4.9, *p* = 0.17). Subgroup analyses of low and high fibrosis and presence or absence of steatosis on TE with revised RR and SOCRATES‐8A did not yield significant associations.

### 3.3 Liver Steatosis and Fibrosis Outcomes

TE results and proportions of fibrosis stages and steatosis grades are outlined in Figure S7. We found that 8/23 (35%) had steatosis grade ≥ 2 and of cirrhosis was detected in 4/23 (17%) with median LSM 34.7 kPa (IQR 26–48.2) (all four with cirrhosis also had grade ≥ 2 steatosis). Overall, median LSM and CAP scores were 5.1 kPa (IQR 3.8–9.4) and 246 dB/m (IQR 221–296), respectively. No patients were discovered to have decompensated liver disease that had been missed during screening.

### 3.4 After Discharge Outcomes

Attempts to contact subjects for follow‐up by phone occurred at several time points. Overall, 10/23 (43%) subjects were contacted successfully: five once, four twice and one four times; median first contact was at 151 days (IQR 90–409). Eight out of the 10 contacted reported abstinence from alcohol at last follow‐up: median abstinence 7 months (IQR 5.5–8.5). Of the remaining two subjects, one reported ongoing binge drinking, and the other had reduced drinking to two beers per day. One of four subjects with cirrhosis referred to hepatology completed the visit. Due to incomplete follow‐up and small numbers, further statistical analyses could not be performed. Seven out of 23 (30%) subjects were unable to be contacted but had data in the electronic health record. Six out of seven (86%) subjects had hospitalizations due to alcohol (three had two, and one had five) at median follow‐up of 274 days (IQR 125–339) (Table S4).

## 4 Discussion

In this pilot study of TE as an OI for patients with AUD, we found significant increases from baseline in readiness for change as measured by the PS of revised RR and SOCRATES‐8A. Although the numerical changes are small, they are significant because the two‐tailed, paired *t*‐testing captured individual preintervention/postintervention changes in two directions: scores could increase or decrease after the intervention, with the latter potentially likely with favorable TE results. To our knowledge, this is the first study to examine and demonstrate readiness for change as a mechanism behind an OI.

OIs have been best studied in smoking cessation, including feedback after spirometry, pregnancy and hospitalization [10]. In one large community study of spirometry for COPD screening as an OI in patients who smoke, 21% stopped smoking after spirometry compared to 12% without spirometry in the ensuing 2–3 years [21]. The rising prevalence of AUD and ALD increases the urgency to develop and leverage new OIs [22]. Our results add mechanistic data to the growing evidence that opportunistic TE performance in patients at risk for ALD is associated with improved alcohol‐associated outcomes, including reduced drinking, lower AUDIT‐C scores and higher abstinence rates [23, 24, 25]. Although validation studies in a wide range of patients with AUD and other substance use disorders have shown that a person's expression of readiness to change behaviour as measured by the RR and SOCRATES‐8A is related to actual change, our study was unable to show this due to loss to follow‐up and small numbers as a pilot study [18, 26, 27, 28, 29, 30, 31].

We found that the ‘recognition’ and ‘taking steps’ domains of SOCRATES‐8A increased after TE as an OI. The ‘recognition domain’ reflects the extent to which respondents acknowledge they are experiencing an alcohol problem and perceive that harm will come if they do not change [32]. Quantitative results of TE with scripted interpretation directly informed subjects about the impact of alcohol on their liver health. The ‘taking steps’ domain reflects the extent to which respondents feel they are actively making positive changes in their drinking. Study participation and TE may have provided additional motivation to subjects admitted to an addiction unit. The ‘ambivalence’ domain reflects the extent to which the respondents are conflicted about the pros and cons of their drinking. ‘Ambivalence’ domain scores did not change after TE as an OI, including subanalyses of low and high fibrosis groups, which would likely have the highest chance of altering this domain.

The HAIS scores were unaffected by TE as an OI. This may be due to several factors, including lack of validation as a repeated test in the same session or in countries outside of Korea, as well as a lack of sensitivity to detect smaller changes in insight. HAIS may not have changed because it is not a ‘liver‐centric’ measurement, and most of its insight‐related components would not be expected to change with new awareness of current or future risk of liver disease. Future studies may benefit from inclusion of a new psychometric tool for alcohol‐associated insight developed by investigators at Henry Ford Health, which was created specifically for use in hepatology [33]. Notably, the theoretical concern that favourable TE results could worsen readiness to change alcohol use was unfounded. One concern was that normal TE results could falsely reassure patients that their liver was immune to damage from alcohol use. This was not detected on psychometric testing and supports truthfulness when discussing liver health to at‐risk patients with AUD [34]. These results suggest that TE may have an expanded role in the care of at‐risk patients beyond a diagnostic test: as a therapeutic, patient‐centred OI (theranostic) that provides understanding of ALD and promotes healthy change. TE as an OI could be a key addition to the integrated, comprehensive medical care experience for patients with AUD by bringing a rapid and reliable bedside test to the patient combined with psychosocial interventions and pharmacotherapy. It has been suggested that liver transplantation serves as a motivator for abstinence due to external pressures, constant reminders of the consequences of heavy alcohol use and new identity as a transplant patient, which may help to command change [35]. Using the visual of the liver on TE as an instrument to demonstrate the ability of the liver to regenerate itself and become new again may empower the patient to do likewise, increasing motivation to command change.

Strengths of this study include its prospective design with validated PS to examine potential mechanisms underlying TE as an OI. We also minimized overestimations of liver stiffness in patients with recent alcohol use and liver inflammation by adjusting for liver tests on admission [20]. Limitations of this study include its small sample size, which prevented subgroup analyses and lack of follow‐up with alcohol biomarker confirmation. As a pilot study assessing feasibility, the study lacked a control group, and patient recruitment was based on author convenience (not consecutive enrolment). Future studies of TE as an OI in a randomized controlled cohort of outpatients with AUD would be informative.

## 5 Conclusion

In this pilot study, we demonstrate that performing and interpreting results of TE as an OI to inpatients with AUD at risk of liver disease increases readiness for change and efficiently detects advanced fibrosis.

## Author Contributions

**Stephanie M. Rutledge:** study design, subject enrollment, data collection, data analysis, writing – original draft. **Rohit R. Nathani:** subject enrollment, data collection, data analysis, writing – original draft. **Patricia Miguez Arosemena:** subject enrollment, data collection. **Daniel Suter:** subject identification, data collection. **David Lehman:** subject identification, data collection. **Timothy Brennan:** study design, subject identification. **Gene Y. Im:** study design, data collection, data analysis, writing – original draft (equal).

## Conflicts of Interest

The authors declare no conflicts of interest.

## Supporting information


**Figure S1.** Hanil Alcohol Insight Scale (HAIS).
**Figure S2.** Revised Readiness Ruler.
**Figure S3.** Stages Of Change Readiness And Treatment Eagerness Scale (SOCRATES‐8A).
**Figure S4.** Domains and Scoring of Stages Of Change Readiness And Treatment Eagerness Scale (SOCRATES‐8A).
**Figure S5.** Alcohol Use Disorders Identification Test, Consumption (AUDIT‐C).
**Figure S6.** CONSORT flowchart.
**Figure S7.** Prevalence of Fibrosis and Steatosis by Transient Elastography.
**Table S1.** Scripted Responses according to Transient Elastography Results.
**Table S2.** Liver Stiffness Measurement Adjustment based on Liver Tests.
**Table S3.** Study Criteria.
**Table S4.** Post‐discharge Outcomes.

## Data Availability

The data that support the findings of this study are available from the corresponding author upon reasonable request.

## References

[1] P. D. Axley , C. T. Richardson , and A. K. Singal , “Epidemiology of Alcohol Consumption and Societal Burden of Alcoholism and Alcoholic Liver Disease,” Clinics in Liver Disease 23, no. 1 (2019): 39–50. 30454831 10.1016/j.cld.2018.09.011

[2] R. J. Wong and A. K. Singal , “Trends in Liver Disease Etiology Among Adults Awaiting Liver Transplantation in the United States, 2014–2019,” JAMA Network Open 3, no. 2 (2020): e1920294–e1920294. 32022875 10.1001/jamanetworkopen.2019.20294 PMC12124732

[3] B. B. Duncan , M. I. Schmidt , and Collaborators GBoDC , “The Global, Regional, and National Burden of Cirrhosis by Cause in 195 Countries and Territories, 1990–2017: A Systematic Analysis for the Global Burden of Disease Study 2017,” Lancet Gastroenterology & Hepatology Amsterdam 5, no. 2020 (2020): 245–266. 10.1016/S2468-1253(19)30349-8 PMC7026710 31981519

[4] S. Spillane , M. S. Shiels , A. F. Best , et al., “Trends in Alcohol‐Induced Deaths in the United States, 2000–2016,” JAMA Network Open 3, no. 2 (2020): e1921451–e1921451. 32083687 10.1001/jamanetworkopen.2019.21451 PMC7043198

[5] M. S. Pollard , J. S. Tucker , and H. D. Green , “Changes in Adult Alcohol use and Consequences During the COVID‐19 Pandemic in the US,” JAMA Network Open 3, no. 9 (2020): e2022942. 32990735 10.1001/jamanetworkopen.2020.22942 PMC7525354

[6] E. B. Tapper and N. D. Parikh , “Mortality due to Cirrhosis and Liver Cancer in the United States, 1999–2016: Observational Study,” BMJ 362 (2018): k2817, 10.1136/bmj.k2817. 30021785 PMC6050518

[7] G. Addolorato , A. Mirijello , P. Barrio , and A. Gual , “Treatment of Alcohol Use Disorders in Patients With Alcoholic Liver Disease,” Journal of Hepatology 65, no. 3 (2016): 618–630, 10.1016/j.jhep.2016.04.029. 27155530

[8] A. Khan , A. Tansel , D. L. White , et al., “Efficacy of Psychosocial Interventions in Inducing and Maintaining Alcohol Abstinence in Patients With Chronic Liver Disease: A Systematic Review,” Clinical Gastroenterology and Hepatology 14, no. 2 (2016): 191–202.e1–4; quiz e20, 10.1016/j.cgh.2015.07.047. 26256464 PMC4805368

[9] C. Keyworth , T. Epton , J. Goldthorpe , R. Calam , and C. J. Armitage , “Delivering Opportunistic Behavior Change Interventions: A Systematic Review of Systematic Reviews,” Prevention Science 21, no. 3 (2020): 319–331, 10.1007/s11121-020-01087-6. 32067156 PMC7056685

[10] C. M. McBride , K. M. Emmons , and I. M. Lipkus , “Understanding the Potential of Teachable Moments: The Case of Smoking Cessation,” Health Education Research 18, no. 2 (2003): 156–170, 10.1093/her/18.2.156. 12729175

[11] P. J. Dillon , S. K. Kedia , O. O. Isehunwa , and M. Sharma , “Motivations for Treatment Engagement in a Residential Substance Use Disorder Treatment Program: A Qualitative Study,” Substance Abuse: Research and Treatment 14 (2020): 1178221820940682, 10.1177/1178221820940682. 32922019 PMC7457698

[12] C. C. DiClemente , L. E. Bellino , and T. M. Neavins , “Motivation for Change and Alcoholism Treatment,” Alcohol Research & Health 23, no. 2 (1999): 86–92. 10890801 PMC6760428

[13] A. B. Laudet and V. Stanick , “Predictors of Motivation for Abstinence at the end of Outpatient Substance Abuse Treatment,” Journal of Substance Abuse Treatment 38, no. 4 (2010): 317–327, 10.1016/j.jsat.2010.01.007. 20185267 PMC2859988

[14] L. Shaul , M. Blankers , M. W. J. Koeter , G. M. Schippers , and A. E. Goudriaan , “The Role of Motivation in Predicting Addiction Treatment Entry Among Offenders With Substance Use Disorders Under Probation Supervision,” International Journal of Offender Therapy and Comparative Criminology 63, no. 14 (2019): 2453–2465, 10.1177/0306624x19849554. 31088187

[15] J. Rivest , D. Jutras‐Aswad , and P. A. Shapiro , “Treating the “Unhealthy Alcohol User” on Medical Wards: Beyond Withdrawal,” Journal of Psychiatric Practice 19, no. 3 (2013): 213–226. 23653078 10.1097/01.pra.0000430505.52391.48

[16] R. K. Sterling , A. Duarte‐Rojo , K. Patel , et al., “AASLD Practice Guideline on Imaging‐Based Noninvasive Liver Disease Assessment of Hepatic Fibrosis and Steatosis,” Hepatology 81, no. 2 (2025): 672–724, 10.1097/hep.0000000000000843. 38489518

[17] J. S. Kim , G. J. Kim , J. M. Lee , C. S. Lee , and J. K. Oh , “HAIS (Hanil Alcohol Insight Scale): Validation of an Insight‐Evaluation Instrument for Practical Use in Alcoholism,” Journal of Studies on Alcohol 59, no. 1 (1998): 52–55, 10.15288/jsa.1998.59.52. 9498315

[18] M. Hesse , “The Readiness Ruler as a Measure of Readiness to Change Poly‐Drug Use in Drug Abusers,” Harm Reduction Journal 3 (2006): 3, 10.1186/1477-7517-3-3. 16436208 PMC1395301

[19] W. R. Miller and J. S. Tonigan , “Assessing Drinkers' Motivation for Change: The Stages of Change Readiness and Treatment Eagerness Scale (SOCRATES),” Psychology of Addictive Behaviors 10 (1996): 81–89.

[20] E. Nguyen‐Khac , M. Thiele , C. Voican , et al., “Non‐Invasive Diagnosis of Liver Fibrosis in Patients With Alcohol‐Related Liver Disease by Transient Elastography: An Individual Patient Data Meta‐Analysis,” Lancet Gastroenterology & Hepatology 3, no. 9 (2018): 614–625. 29983372 10.1016/S2468-1253(18)30124-9

[21] N. G. Hepper , C. W. Drage , S. F. Davies , et al., “Chronic Obstructive Pulmonary Disease: A Community‐Oriented Program Including Professional Education and Screening by a Voluntary Health Agency,” American Review of Respiratory Disease 121, no. 1 (1980): 97–104, 10.1164/arrd.1980.121.1.97. 7352718

[22] M. Mitka , ““Teachable Moments” Provide a Means for Physicians to Lower Alcohol Abuse,” JAMA 279, no. 22 (1998): 1767–1768, 10.1001/jama.279.22.1767. 9628694

[23] E. Avitabile , J. Gratacós‐Ginès , M. Pérez‐Guasch , et al., “Liver Fibrosis Screening Increases Alcohol Abstinence,” JHEP Reports 6, no. 10 (2024): 101165, 10.1016/j.jhepr.2024.101165. 39380719 PMC11459648

[24] M. Subhani , D. G. Enki , H. Knight , et al., “Does Knowledge of Liver Fibrosis Affect High‐Risk Drinking Behaviour (KLIFAD): An Open‐Label Pragmatic Feasibility Randomised Controlled Trial,” EClinicalMedicine 61 (2023): 102069, 10.1016/j.eclinm.2023.102069. 37448808 PMC10336239

[25] M. Kjaergaard , K. P. Lindvig , K. H. Thorhauge , et al., “Screening for Fibrosis Promotes Lifestyle Changes: A Prospective Cohort Study in 4796 Individuals,” Clinical Gastroenterology and Hepatology 22, no. 5 (2024): 1037–1047.e9, 10.1016/j.cgh.2023.12.018. 38154729

[26] S. A. Maisto , M. Krenek , T. Chung , C. S. Martin , D. Clark , and J. Cornelius , “A Comparison of the Concurrent and Predictive Validity of Three Measures of Readiness to Change Alcohol Use in a Clinical Sample of Adolescents,” Psychological Assessment 23, no. 4 (2011): 983–994, 10.1037/a0024136. 21767028 PMC3433156

[27] J. J. Burrow‐Sánchez , C. Corrales , and J. Totsky , “Predictive Validity of the SOCRATES in a Clinical Sample of Latina/o Adolescents,” Psychology of Addictive Behaviors 33, no. 2 (2019): 171–177, 10.1037/adb0000432. 30589309 PMC6405303

[28] K. M. Lillie , K. J. Jansen , L. G. Dirks , et al., “Assessing the Predictive Validity of the Stages of Change Readiness and Treatment Eagerness Scale (SOCRATES) in Alaska Native and American Indian People,” Journal of Addiction Medicine 14, no. 5 (2020): e241–e246, 10.1097/adm.0000000000000661. 32371661 PMC7541407

[29] F. J. Schwebel , J. G. Chavez , and M. R. Pearson , “Measuring Readiness to Change Substance Use, Alcohol Use, and Cannabis Use: An Experimental Manipulation of Cognitive Effort,” Substance Use & Misuse 58, no. 8 (2023): 1062–1068, 10.1080/10826084.2023.2205539. 37139932 PMC10259820

[30] E. C. Williams , N. J. Horton , J. H. Samet , and R. Saitz , “Do Brief Measures of Readiness to Change Predict Alcohol Consumption and Consequences in Primary Care Patients With Unhealthy Alcohol Use?,” Alcoholism, Clinical and Experimental Research 31, no. 3 (2007): 428–435, 10.1111/j.1530-0277.2006.00324.x. 17295727

[31] N. Heather , D. Smailes , and P. Cassidy , “Development of a Readiness Ruler for Use With Alcohol Brief Interventions,” Drug and Alcohol Dependence 98, no. 3 (2008): 235–240, 10.1016/j.drugalcdep.2008.06.005. 18639393

[32] SAMHSA , “Enhancing Motivation for Change in Substance Use Disorder Treatment 2019,” (2019). Accessed 08/05/2024, https://store.samhsa.gov/sites/default/files/tip‐35‐pep19‐02‐01‐003.pdf. 34106565

[33] G. S. Winder , V. Gill , S. Patel , H. Asefa , and J. L. Mellinger , “Expert and Patient Cognitive Interviews in the Development of a Novel Alcohol Insight Scale for Use in Hepatology and Liver Transplantation,” General Hospital Psychiatry 91 (2024): 256–258, 10.1016/j.genhosppsych.2024.09.010. 39317622

[34] G. S. Winder and J. L. Mellinger , “Letter to the Editor: Robust Clinician Relationships Must Be the Bedrock for Future Innovations in Integrated Alcohol‐Associated Liver Disease Care,” Liver Transplantation 30 (2024): E42–E44, 10.1097/lvt.0000000000000403. 38767451

[35] G. E. Vaillant , “The Natural History of Alcoholism and Its Relationship to Liver Transplantation,” Liver Transplantation and Surgery 3, no. 3 (1997): 304–310. 9346756 10.1002/lt.500030318

