# Supplementary material for: Transient Elastography Increases Readiness for Change in Inpatients With Alcohol Use Disorder: The ELISA Pilot Study
Source: Addict Biol. 2025 Jun 23;30(6):e70043. doi: 10.1111/adb.70043 (PMC12184072; doi:10.1111/adb.70043)
Supplement: Supplementary file 1 — Figure S1. Hanil Alcohol Insight Scale (HAIS). Figure S2. Revised Readiness Ruler. Figure S3. Stages Of Change Readiness And Treatment Eagerness Scale (SOCRATES‐8A). Figure S4. Domains and Scoring of Stages Of Change Readiness And Treatment Eagerness Scale (SOCRATES‐8A). Figure S5. Alcohol Use Disorders Identification Test, Consumption (AUDIT‐C). Figure S6. CONSORT flowchart. Figure S7. Prevalence of Fibrosis and Steatosis by Transient Elastography. Table S1. Scripted Responses according to Transient Elastography Results. Table S2. Liver Stiffness Measurement Adjustment based on Liver Tests. Table S3. Study Criteria. Table S4. Post‐discharge Outcomes. [file ADB-30-e70043-s001.docx]

**SUPPLEMENTAL FIGURES AND TABLES**

**Supplemental Figure 1. Hanil Alcohol Insight Scale (HAIS)**


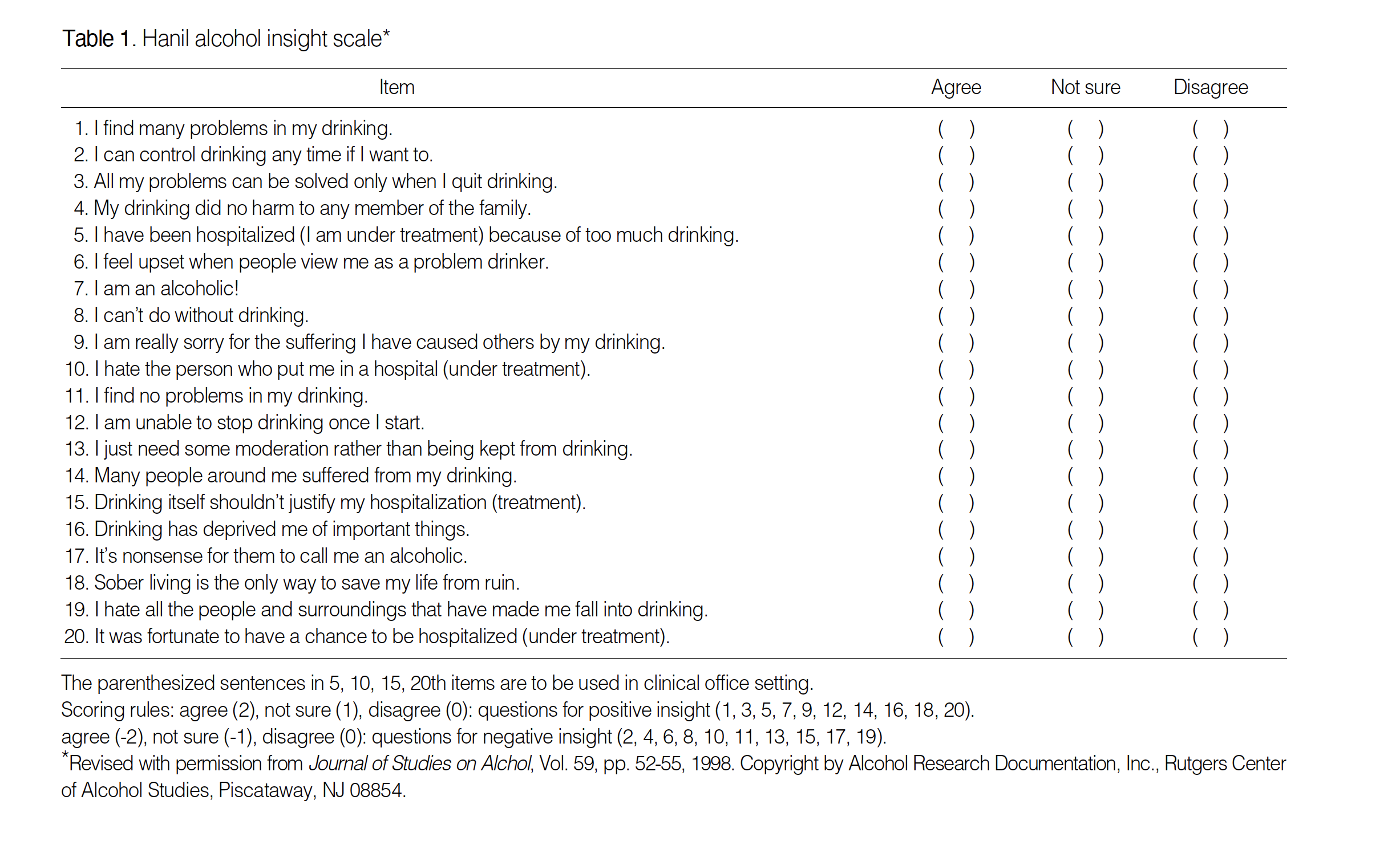


**Supplemental Figure 2. Revised Readiness Ruler**

If your readiness to make a change to your drinking started at 5 on this scale before the test, how do you rate it now?


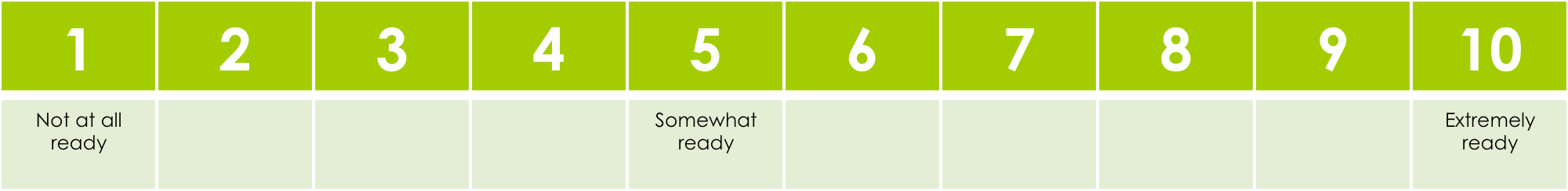


**Supplemental Figure 3. Stages Of Change Readiness And Treatment Eagerness Scale (SOCRATES-8A)**


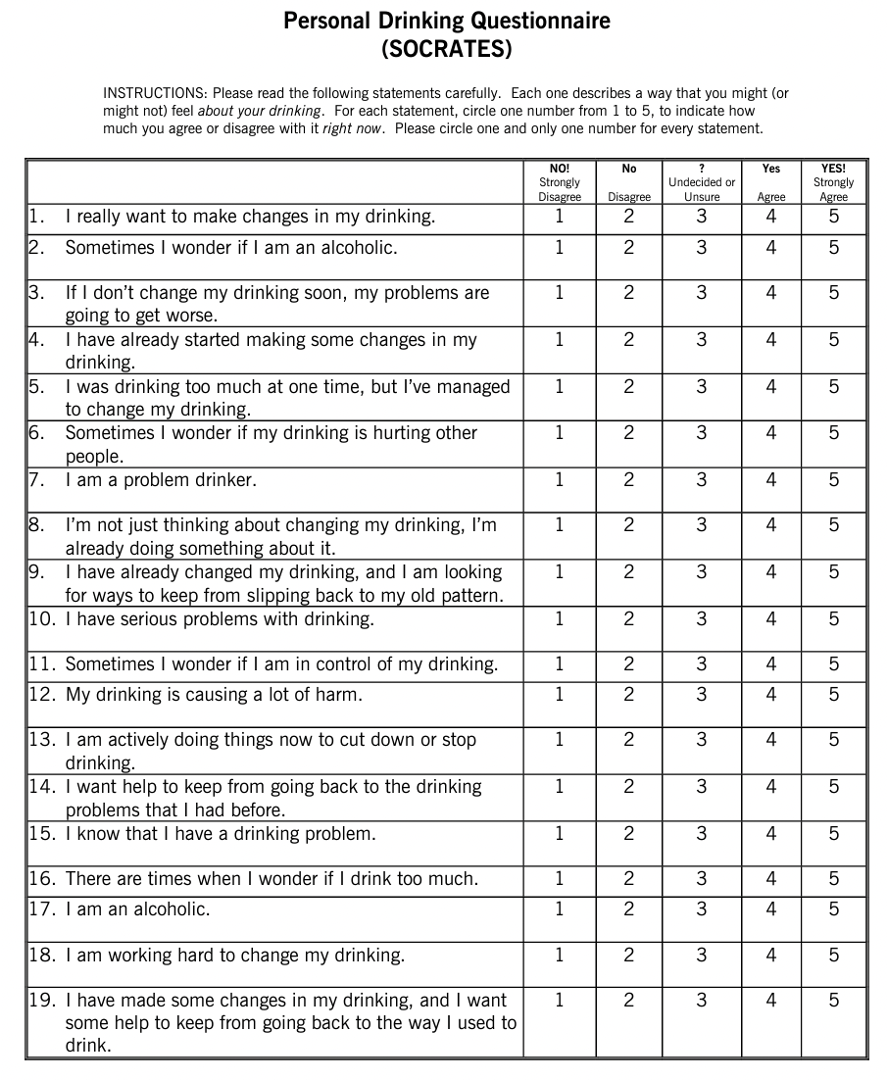


**Supplemental Figure 4. Domains and Scoring of Stages Of Change Readiness And Treatment Eagerness Scale (SOCRATES-8A)**

| **SOCRATES-8A Domains** | **Explanation** |
| --- | --- |
| **Recognition**  (Questions 1, 3, 7, 10, 12, 15, 17) | **High scorers** directly acknowledge that they are having problems related to their drinking, tending to express a desire for change and to perceive that harm will continue if they do not change.  **Low scorers** deny that alcohol is causing them serious problems, reject diagnostic labels such as “problem drinker” and “alcoholic,” and do not express a desire for change. |
| **Ambivalence**  (Questions 2, 6, 11, 16) | **High scorers** say that they sometimes are drinking too much, are hurting other people, and/or are “alcoholic.” Thus a high score reflects ambivalence or uncertainty. A high score here reflects some openness to reflection, as might be particularly expected in the contemplation stage of change.  **Low scorers** say that they do not wonder if they are drinking too much or are hurting others, or are “alcoholic.” Note that a person may score low on ambivalence because they “know” their drinking is causing problems (high Recognition), or “know” that they do not have drinking problems (low Recognition). Thus a low Ambivalence score should be interpreted in relation to the Recognition score. |
| **Taking Steps**  (Questions 4, 5, 8, 9, 13, 14, 18, 19) | **High scorers** report that they are already doing things to make a positive change in their drinking, and may have experienced some success in this regard. Change is under way, and they may want help to persist or to prevent backsliding. A high score on this scale has been found to be predictive of successful change.  **Low scorers** report that they are not currently doing things to change their drinking, and have not made such changes recently. |


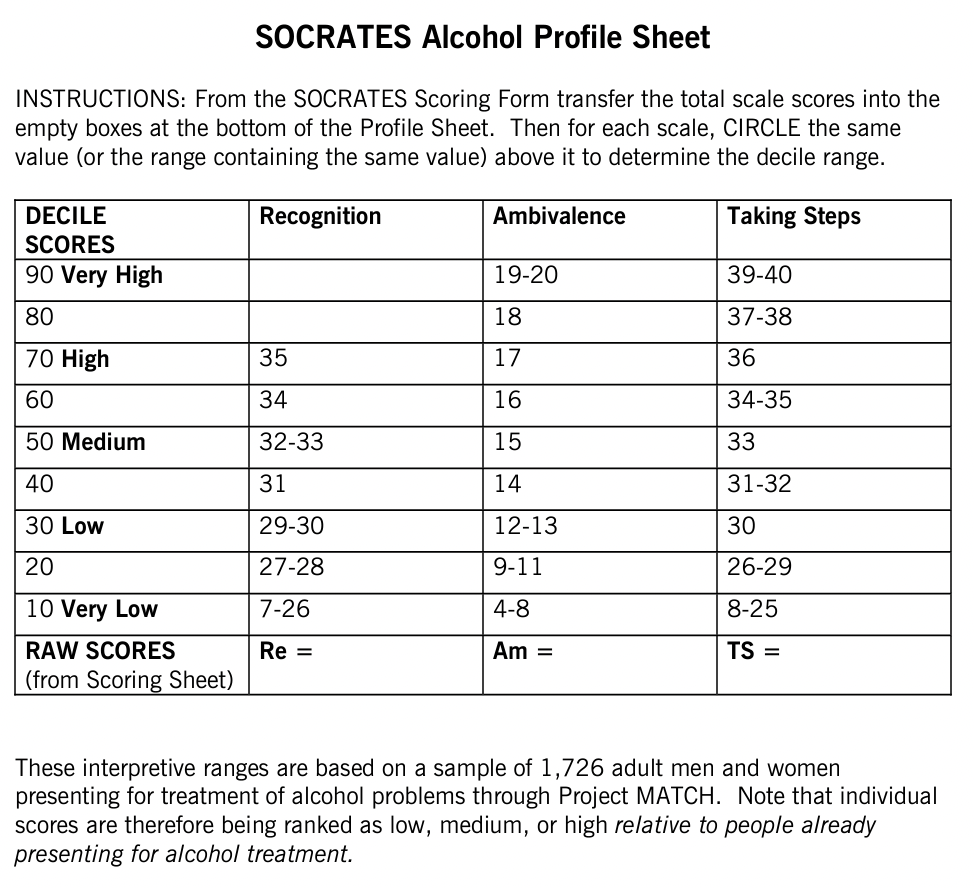


**Supplemental Figure 5. Alcohol Use Disorders Identification Test, Consumption (AUDIT-C)**


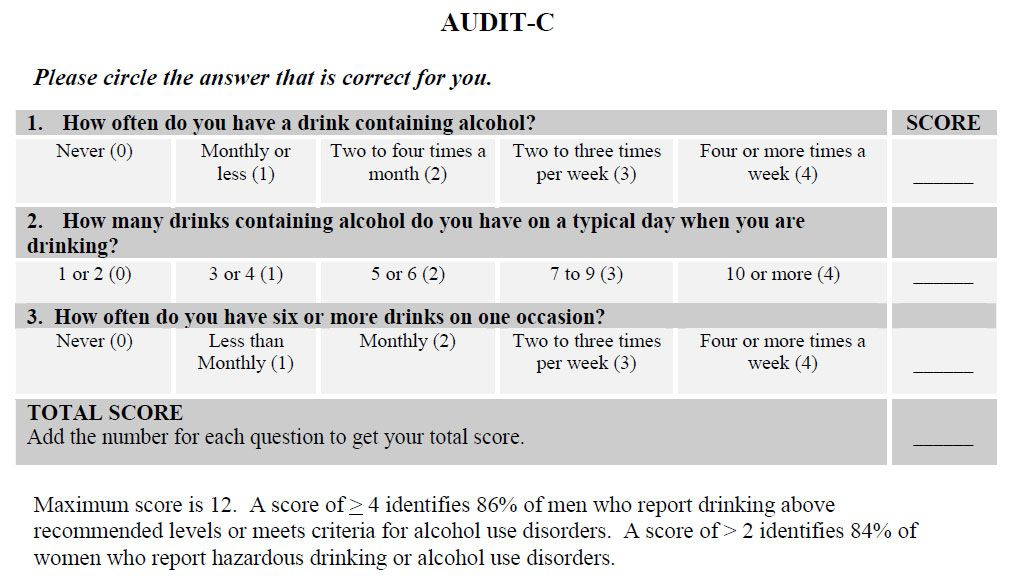


**Supplemental Figure 6. CONSORT flowchart**

**Supplemental Figure 7. Prevalence of Fibrosis and Steatosis by Transient Elastography**

**
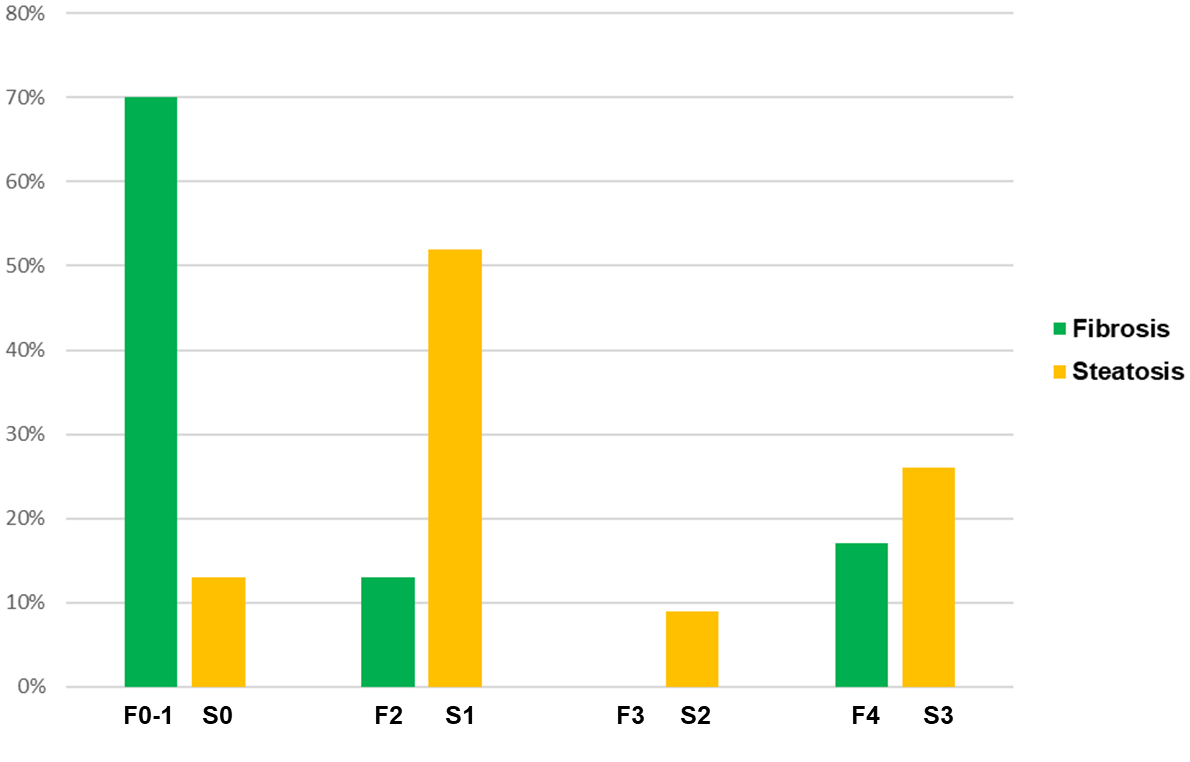
**

**Supplemental Table 1. Scripted Responses according to Transient Elastography Results**

| **Fibrosis** | |
| --- | --- |
| **Fibrosis Stage 0** | “The results of the liver scan suggest that you have no liver scarring. This test is not perfect but is a sign that there has been no long-term damage done to your liver by alcohol. This is good news but it does not mean that you are protected from future damage to your liver.” |
| **Fibrosis Stage 1** | “The results of the liver scan suggest that you have mild liver scarring. This test is not perfect and can be wrong up to 20% of the time. However, this is a sign that there has been only mild damage done to your liver by alcohol. This is good news, but does not mean that you are protected from future damage to your liver.” |
| **Fibrosis Stage 2** | “The results of the liver scan suggest that you have moderate liver scarring. This test is not perfect and can be wrong up to 20% of the time. However, this result is a sign that there has been some damage done to your liver by alcohol. As this is a research study, we don’t have the ability to investigate further but we would recommend that you see a liver doctor in the clinic and we can help with that.” |
| **Fibrosis Stage 3** | “The results of the liver scan suggest that you have severe liver scarring. This test is not perfect and can be wrong up to 20% of the time. However, this is a sign that there has been significant damage done to your liver by alcohol. As this is a research study, we don’t have the ability to investigate further but we would recommend that you see a liver doctor in the clinic and we can help with that.” |
| **Fibrosis Stage 4** | “The results of the liver scan suggest that you have advanced liver scarring (cirrhosis). This test is not perfect and can be wrong up to 10% of the time. However, this is a sign that there has been significant damage done to your liver by alcohol. As this is a research study, we don’t have the ability to investigate further but we would recommend that you see a liver doctor in the clinic and we can help with that.” |

| **Steatosis** | |
| --- | --- |
| **Steatosis Grade 0-1** | “The results of the liver scan show that you have a none to a small amount of fatty change in the liver. This is mild and does not usually cause any liver problems, but could be linked to liver disease in the future.” |
| **Steatosis Grade 2** | “The results of the liver scan show that you have a moderate amount of fatty change in the liver. With fibrosis, this can progress to more significant liver disease in the future.” |
| **Steatosis Grade 3** | “The results of the liver scan show that you have a high amount of fatty change in the liver. With fibrosis, this can progress to more significant liver disease in the future.” |

**Supplemental Table 2. Liver Stiffness Measurement Adjustment based on Liver Tests**

| **Liver Tests** | **Adjusted Liver Stiffness Measurement Thresholds** |
| --- | --- |
| AST <38.7  and  TB <0.5 | <5.6 kPa = F0 fibrosis  ≥5.6 kPa = F1 fibrosis  ≥6.9 kPa = F2 fibrosis  ≥8.8 kPa = F3 fibrosis  ≥12.1 kPa = F4 fibrosis |
| AST 38.7-75 and  TB <0.5  or  AST <38.7  and  TB 0.5-0.9 | <6.9 kPa = F0 fibrosis  ≥6.9 kPa = F1 fibrosis  ≥8.1 kPa = F2 fibrosis  ≥11.2 kPa = F3 fibrosis  ≥15.4 kPa = F4 fibrosis |
| AST 38.7-75  and  TB 0.5-0.9 | <8.4 kPa = F0 fibrosis  ≥8.8 kPa = F1 fibrosis  ≥8.8 kPa = F2 fibrosis  ≥12.3 kPa = F3 fibrosis  ≥19.9 kPa = F4 fibrosis |
| AST >75  and  TB >0.9 | <9.6 kPa = F0 fibrosis  ≥9.6 kPa = F1 fibrosis  ≥11.6 kPa = F2 fibrosis  ≥16.1 kPa = F3 fibrosis  ≥25.9 kPa = F4 fibrosis |

Abbreviations: AST, aspartate aminotransferase; TB, total bilirubin

AST expressed as IU/L, TB expressed as mg/dL

**Supplemental Table 3. Study Criteria**

| Inclusion criteria | Patients aged 18 and above with a diagnosis of AUD according to DSM-V criteria without known liver disease or prior TE who were admitted to the inpatient addiction center at Mount Sinai West Hospital. |
| --- | --- |
| Exclusion criteria | Prior TE in the past 1-year, uncontrolled hepatic encephalopathy, uncontrolled concomitant psychiatric disorder, revised clinical institute withdrawal assessment for alcohol scale (CIWA-AR) score >20 (severe alcohol withdrawal symptoms), or an inability to perform TE due to body habitus, ascites, or technical issues. Pregnant women, people with battery-operated, implanted cardiac devices or those who were deemed unfit to fast for 3 hours before TE were excluded. |

Abbreviations: AUD, alcohol use disorder; DSM-V, Diagnostic and Statistical Manual of Mental Disorders, Fifth Edition; TE, transient elastography.

**Supplemental Table 4. Post-discharge Outcomes.**

| **Outcome** | **n (%) or n (IQR)** |
| --- | --- |
| *Follow-up contact attempts* |  |
| Successfully contacted | 10/23 (43%)* |
| Median time to first contact (days) | 151 (90-409) |
| *Self-reported alcohol use* |  |
| Reported abstinence | 8/10 (80%) |
| Median duration of abstinence (months) | 7 (5.5-8.5) |
| Ongoing binge drinking | 1/10 (10%) |
| Reduced drinking | 1/10 (10%) |
| *Follow-up for advanced fibrosis* |  |
| Completed hepatology follow-up | 1/4 (25%) |
| *Electronic health record data* |  |
| Unable to be contacted but had EHR data | 7/23 (30%) |

*5 contacted once, 4 twice, 1 four times

**2 patients had 1 hospitalization, 3 had two, 1 had five

Abbreviations:

EHR: Electronic health record
